# Effective immunosuppression with dexamethasone phosphate in the *Galleria mellonella* larva infection model resulting in enhanced virulence of *Escherichia coli* and *Klebsiella pneumoniae*

**DOI:** 10.1007/s00430-016-0450-5

**Published:** 2016-02-26

**Authors:** Miquel Perez Torres, Frances Entwistle, Peter J. Coote

**Affiliations:** Biomedical Sciences Research Complex, School of Biology, University of St Andrews, The North Haugh, St Andrews, Fife KY16 9ST UK; Department of Microbiology, Facultad de Biología, University of Barcelona, Diagonal, 643, 08028 Barcelona, Spain

**Keywords:** Insect infection model, Antibacterial, Antimicrobial, Ceftazidime, Pathogenicity, Glucocorticoid anti-inflammatory

## Abstract

The aim was to evaluate whether immunosuppression with dexamethasone 21-phosphate could be applied to the *Galleria mellonella* in vivo infection model. Characterised clinical isolates of *Escherichia coli* or *Klebsiella pneumoniae* were employed, and *G. mellonella* larvae were infected with increasing doses of each strain to investigate virulence in vivo. Virulence was then compared with larvae exposed to increasing doses of dexamethasone 21-phosphate. The effect of dexamethasone 21-phosphate on larval haemocyte phagocytosis in vitro was determined via fluorescence microscopy and a burden assay measured the growth of infecting bacteria inside the larvae. Finally, the effect of dexamethasone 21-phosphate treatment on the efficacy of ceftazidime after infection was also noted. The pathogenicity of *K. pneumoniae* or *E. coli* in *G. mellonella* larvae was dependent on high inoculum numbers such that virulence could not be attributed specifically to infection by live bacteria but also to factors associated with dead cells. Thus, for these strains, *G. mellonella* larvae do not constitute an ideal infection model. Treatment of larvae with dexamethasone 21-phosphate enhanced the lethality induced by infection with *E. coli* or *K. pneumoniae* in a dose- and inoculum size-dependent manner. This correlated with proliferation of bacteria in the larvae that could be attributed to dexamethasone inhibiting haemocyte phagocytosis and acting as an immunosuppressant. Notably, prior exposure to dexamethasone 21-phosphate reduced the efficacy of ceftazidime in vivo. In conclusion, demonstration of an effective immunosuppressant regimen can improve the specificity and broaden the applications of the *G. mellonella* model to address key questions regarding infection.

## Introduction

Globally, multidrug-resistant (MDR) Gram-negative bacteria are a major cause of hospital-acquired infections and are considered an urgent public health threat [1]. The majority of these infections are either pneumonia, bloodstream or urinary tract infections and are associated with invasive medical devices or surgical procedures and effect patients that are already debilitated or immunocompromised (reviewed in [2]). With increasing incidence of resistance to carbapenems, the antibiotics of ‘last-resort’, only a limited number of antibiotic treatments are available for some MDR bacteria [3] resulting in high mortality rates [4].

In the USA, the most common MDR bacteria associated with hospital-acquired infections are *Klebsiella pneumoniae* and *Escherichia coli* [1]. MDR strains of these organisms initially acquired a variety of extended-spectrum β-lactamases (ESBLs) that rendered them resistant to cephalosporin antibiotics resulting in increased use of carbapenems. Subsequently, this selected for the acquisition of carbapenemases rendering strains resistant to this class of drugs also. In the UK, the first strain of *K. pneumoniae* with resistance to carbapenems due to expression of the *K. pneumoniae* carbapenemase 3 enzyme (KPC-3) was reported in 2007 [5]. Also in the UK, concern was first raised in 2003 that MDR *E. coli* expressing the CTX-M-15 ESBL were increasingly being identified in patients with community-acquired urinary tract infections that had no previous history of hospitalisation [6]. This spread of MDR pathogens into general practice with few treatment options represents a serious threat to public health.

Thus, there is clear need to research and develop new antibiotics and treatment regimens to combat MDR Gram-negative infections. To facilitate this process, the use of animal models to measure efficacy of potential new treatments is an indispensable part of the drug discovery pipeline. Presently, the majority of novel antimicrobials that are initially identified in vitro are then tested for efficacy in vivo using murine infection models. The problems associated with employing mammalian infection models include ethical issues, high costs and their impracticality for large-scale screening of efficacy. Thus, researchers are increasingly using alternative infection models such as invertebrates to circumvent these issues.

Larvae of the greater wax moth *Galleria mellonella* have been employed as an in vivo model in many investigations of bacterial pathogenicity or efficacy of antibiotic treatments (reviewed in [7]). Advantages of *G. mellonella* include: fewer ethical concerns, low cost, precise inoculation of bacteria and/or dosing of drugs, ability to incubate larvae at human body temperature and ease of use with regard to high-throughput screening of novel treatments in vivo. Disadvantages include the reduced complexity of the invertebrate immune system compared to mammals due to the absence of adaptive immunity, the inability to study long-term, chronic infections and the exclusion of pathogens that do not display virulence in the larvae.

*Galleria mellonella* larvae have been used to characterise pathogenicity and virulence of *K. pneumoniae* clinical isolates and strains with mutations in known virulence factor genes [8–10]. Furthermore, *G. mellonella* was used to quantify the efficacy of antibiotics against planktonic or biofilm cultures of MDR strains of *K. pneumoniae* [11]. Similar studies with pathogenic *E. coli* have also been undertaken with *G. mellonella* that have correlated lethality with specific virulence factors in uropathogenic [12, 13] and enteropathogenic isolates [14]. Also, *G. mellonella* was comparable to a murine model for measuring virulence of a range of *E. coli* clinical isolates [15]. Notably, assessment of the inoculum size of *K. pneumoniae* or *E. coli* that was required to induce significant lethality in *G. mellonella* reveals huge variability between bacterial strains but also that the minimum number of bacteria required to cause significant larval death, even with the most virulent strains tested, was much higher with *K. pneumoniae* and *E. coli* compared with that required for another Gram-negative pathogen *Pseudomonas aeruginosa* [16, 17].

In this study, the effect of treating *G. mellonella* larvae with a water-soluble derivative of the immunosuppressant drug dexamethasone on subsequent susceptibility to infection by *K. pneumoniae* and *E. coli* was evaluated. The aims of the study were: (1) to decrease the relatively high inoculum of *K. pneumoniae* or *E. coli* cells required to induce a lethal infection in *G. mellonella* larvae to explore whether immunosuppression could be employed as a viable route to expand use of this model to pathogens that do not display virulence or induce acute infections in the larvae; (2) to mimic the immunosuppressed state that is often a significant factor with human patients that suffer from hospital-acquired infections by *K. pneumoniae* and *E. coli*; and finally (3) to assess the effect of immunosuppression on the efficacy of antibiotic treatment for *K. pneumoniae* or *E. coli* infection in vivo.

## Materials and methods

### Bacteria and growth media

Bacteria were obtained from the National Collection of Type Cultures (NCTC) (http://www.hpacultures.org.uk/collections/nctc.jsp). The *K. pneumoniae* strains were NCTC9633 (type strain) and NCTC13438 (a MDR isolate possessing the KPC-3 carbapenemase; [5]), and the *E. coli* strains were NCTC12241 (a control strain for antibiotic sensitivity testing) and NCTC13353 (an isolate possessing the CTX-M-15 ESBL; [6]). All of these strains are clinical isolates. Strains were cultured overnight in Mueller–Hinton Broth (MHB; Merck, Darmstadt, Germany) at 37 °C with shaking to prepare inocula for in vivo infections.

### Reagents and *G. mellonella* larvae

Unless stated, all chemicals and ceftazidime hydrate were purchased from Sigma-Aldrich Ltd (Dorset, UK). Stock solutions and sub-stocks of all drugs for use in experiments were made in sterile deionised water. *G. mellonella* larvae were purchased from UK Waxworms Ltd (Sheffield, UK).

### *Galleria mellonella* infection model and treatment with dexamethasone

This was carried out as previously described [17]. Briefly, groups of 15 larvae were infected with variable inoculum sizes of *K. pneumoniae* or *E. coli* cells and incubated at 37 °C. Larval survival was determined every 24 h for 96 h in total. Precise numbers of bacterial cells inoculated were determined by serial dilution in MHB and plating on Nutrient Agar (NA; Merck, Darmstadt, Germany) prior to counting colonies after overnight incubation of plates at 37 °C. Each experiment was repeated at least twice using larvae from different batches. The data from these replicate experiments were then pooled, and survival data were plotted using the Kaplan–Meier method and comparisons made between groups using the log-rank test. In all comparisons to the negative control, it was the uninfected control (rather than the unmanipulated control) that was used. In all tests, *p* ≤ 0.05 was considered significant and Holm’s correction was applied to account for multiple comparisons [18]. Heat-killed (98 °C, 15 min) inocula at the highest inoculum sizes used were also injected as controls. Heat-killing was verified by plating the heated inocula on NA. To identify any secreted bacterial virulence factors both heat-killed and live inocula were sterile-filtered using Millex 0.22 μM syringe-driven filters (Millipore Ltd, Tullagren, Co. Cork, Ireland) and the filtrate injected into the larvae.

The effect of exposure to dexamethasone was tested by injecting dexamethasone 21-phosphate disodium (50 mg/mL stock solution in H_2_O) for variable time periods (10 min, 2, 4, 6 and 8 h) or at variable doses (80 or 200 μg per larva) prior to infection of larvae with bacteria.

For experiments involving a single dose of ceftazidime, the antibiotics were administered 2 h post-infection (p.i).

### *Galleria mellonella* haemolymph burden

Groups of 40 larvae were treated with 200 μg per larva of dexamethasone 21-phosphate disodium prior to infection with known numbers of *K. pneumoniae* or *E. coli* cells and incubation at 37 °C. After 5 h incubation, and every 24 h thereafter, five larvae were selected at random from each treatment group and tested for haemolymph burden exactly as previously described [19].

### Phagocytosis assays

The method was adapted from Mandato et al. [20]. Larvae were chilled at 4 °C for 15 min prior to injection with 75 μL of *Galleria* saline (GS; 186 mM NaCl, 13 mM KCl, 10 mM HEPES, 1 mM NaHCO_3_, 2 mM CaCl_2_; pH 6.8) saturated with phenylthiol urea (PTU). Haemolymph was collected from 5 larvae per assayed treatment via amputation of the terminal thoracic prolegs. The haemolymph was centrifuged 5 min at 500 g and the haemocytes resuspended in GIM (Grace’s Insect Medium) saturated with PTU and dexamethasone 21-phosphate disodium at 5 mg/mL or water for the untreated control. Haemocyte viability was measured by counting haemocytes that exclude Trypan blue (0.02 % in PBS) using a haemocytometer exactly as described previously [20]. 250 μL of this suspension was placed in separate poly (2-hydroxyethyl methacrylate)-coated wells in 24-well culture plates (Cellstar^®^, Greiner bio-one, Frickenhausen, Germany). After 15 min, inactivated fluorescein (FITC)-labelled *E. coli* K12 Bioparticles^®^ (ThermoFisher Scientific, Waltham, MA, USA), 1.0 × 10^8^ lyophilised cells per mL resuspended in GS + PTU, were added and incubated at 37 °C for 1 and 2 h with gentle agitation. Haemocytes were quantified using a haemocytometer, and the number of fluorescent bacteria added was calculated such that that the proportion of bacteria to haemocytes was approximately 50:1. The assay was stopped by addition of 1 mL of 3.7 % formaldehyde in GS. The fixed haemocytes were then pelleted at 500 g for 4 min and washed with GS to remove excess fluorescent bacteria prior to a 1:1 dilution in glycerol and dispensing on glass microscope slides. Haemocytes and fluorescent *E. coli* were viewed at 40× magnification using a Delta Vision microscope (Applied Precision, Issaquah, Wash., USA) using differential interference contrast and filters for FITC (excitation 490 nm/emission 528 nm). Composite images were captured of optical slices through each haemocyte, and internalised fluorescent bacteria were counted. For each time point tested, phagocytosed *E. coli* from at least 30 haemocytes, from at least two replicate experiments, were counted.

## Results

### The pathogenicity of some clinical isolates of *K. pneumoniae* or *E. coli* in *G. mellonella* larvae is dependent on high inoculum numbers and cannot be attributed specifically to infection by live bacteria

**Fig. 1:**
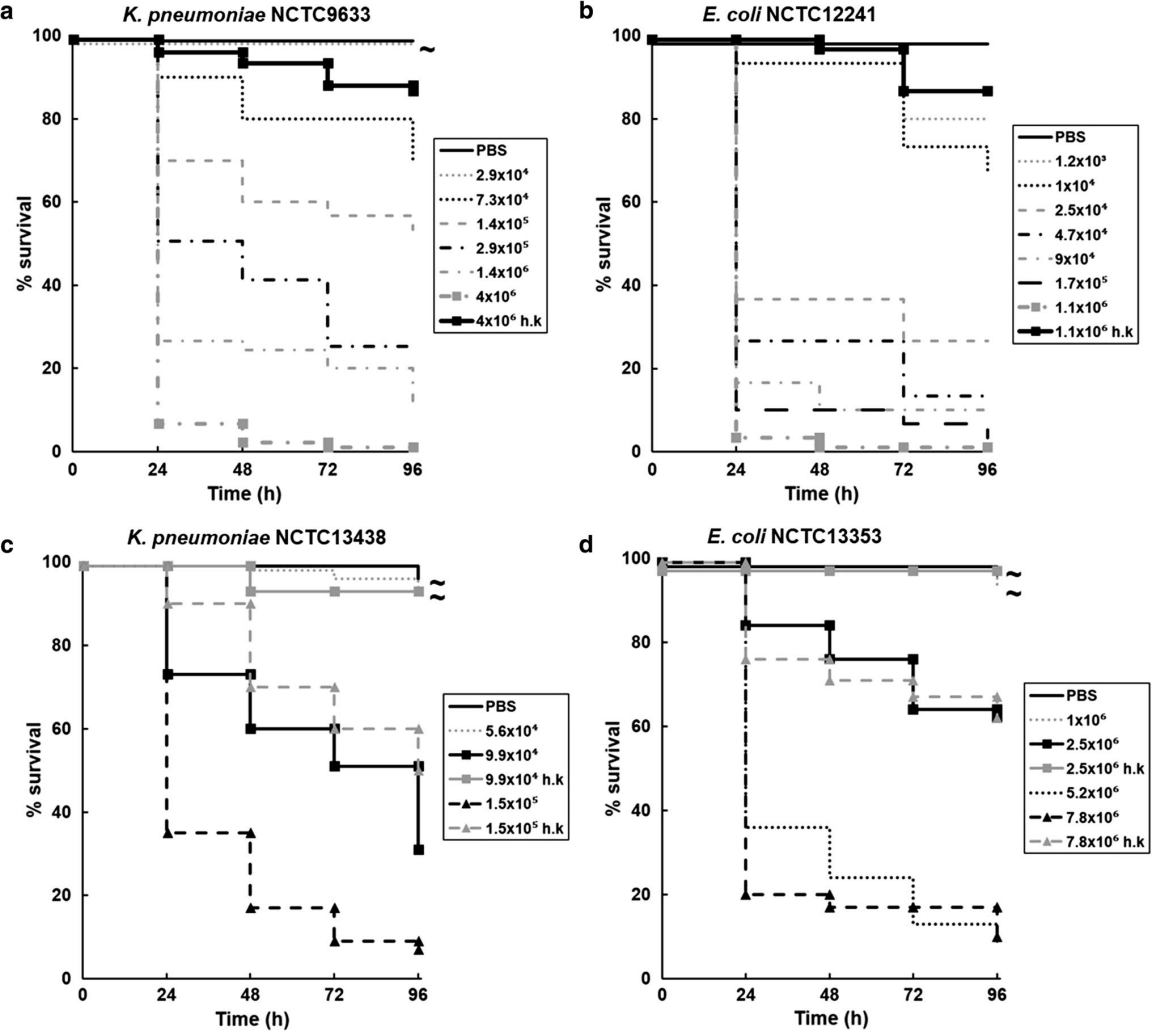
Effect of increasing inoculum dose of live *K. pneumoniae* NCTC9633 (**a**) or NCTC13438 (**c**) and *E. coli* NCTC12241 (**b**) or NCTC13353 (**d**) on the survival of *G. mellonella* larvae during incubation at 37 °C for 96 h. Numbers in the legend indicate the inoculum size in cells per larva. For all strains, the effect of heat-killed (h.k.) bacterial inocula is also shown. No significant mortality was observed in an unmanipulated group (data not shown) or in the uninfected group sham-infected with sterile PBS. With all treatment groups *not* labelled with *tilde*, survival was significantly reduced compared to the group sham-infected with PBS (*p* < 0.05, log-rank test with Holm correction for multiple comparisons); *n* = 45 (pooled from replicate experiments)

The effect of infection with *K. pneumoniae* and *E. coli* strains on survival of *G. mellonella* is shown in Fig. 1. There was variation between all the strains in the inoculum size required to kill the majority of the larval population, but, supporting previous studies [8–15], the inoculum size required to kill the majority of infected larvae 24 h post-infection was comparable to that reported for different strains of the same organisms. For all bacterial strains, larval survival over 96 h was reduced in a dose-dependent manner as the inoculum numbers increased. Notably, infection with heat-killed inocula of each strain at the highest dose tested induced variable lethality. For example, infection with either heat-killed *K. pneumoniae* NCTC9633 (4.0 × 10^6^ per larva) or *E. coli* NCTC12241 (1.1 × 10^6^ per larva) killed approximately 10 % of the population after 96 h, whereas heat-killed *K. pneumoniae* NCTC13438 (1.5 × 10^5^ per larva) or *E. coli* NCTC13353 (7.8 × 10^6^ per larva) killed approximately 50 and 40 %, respectively, after 96 h (Fig. 1). To investigate this further, identical heat-killed inocula of *K. pneumoniae* NCTC13438 and *E. coli* NCTC13353 that were subsequently filter-sterilised and then injected into larvae had no detrimental effect on larval survival (data not shown; *p* > 0.05, log-rank test). Similarly, viable inocula of both strains that were filter-sterilised prior to injection also had no detrimental effect on larval survival (data not shown; *p* > 0.05, log-rank test). These results indicate that larval death induced by the heat-killed cells was due to the presence of these dead cells and not because of any secreted, or released, virulence factors present in the medium.

In conclusion, at the high inoculum levels of *K. pneumoniae* NCTC13438 and *E. coli* NCTC13353 required to kill greater than 90 % of the larval population, bacterial pathogenesis cannot be attributed solely to infection by live bacteria because some toxicity is induced simply by the presence of an unknown virulence factor(s) associated with dead cells. Thus, for these particular bacterial strains, *G. mellonella* larvae do not constitute an ideal infection model.

### Treatment with dexamethasone 21-phosphate inhibits phagocytosis and sensitises *G. mellonella* larvae to infection by *K. pneumoniae* or *E. coli*

Dexamethasone is a glucocorticoid anti-inflammatory drug that is used extensively in human medicine. Research has shown that dexamethasone also has immunomodulatory activity in *G. mellonella* larvae by inhibiting phagocytosis in vitro and prophenoloxidase activation in vivo [20]. Mandato et al. [20] administered water insoluble dexamethasone dissolved in ethanol to larvae. Here, the effect of dexamethasone 21-phosphate, the soluble form of the drug used in human medicine, was measured to determine whether it too could induce immunosuppression in *G. mellonella* larvae and whether it could be used to reduce the inoculum size of *E. coli* or *K. pneumoniae* required to cause significant lethality, thus improving the specificity of this infection model for these organisms.

**Fig. 2:**
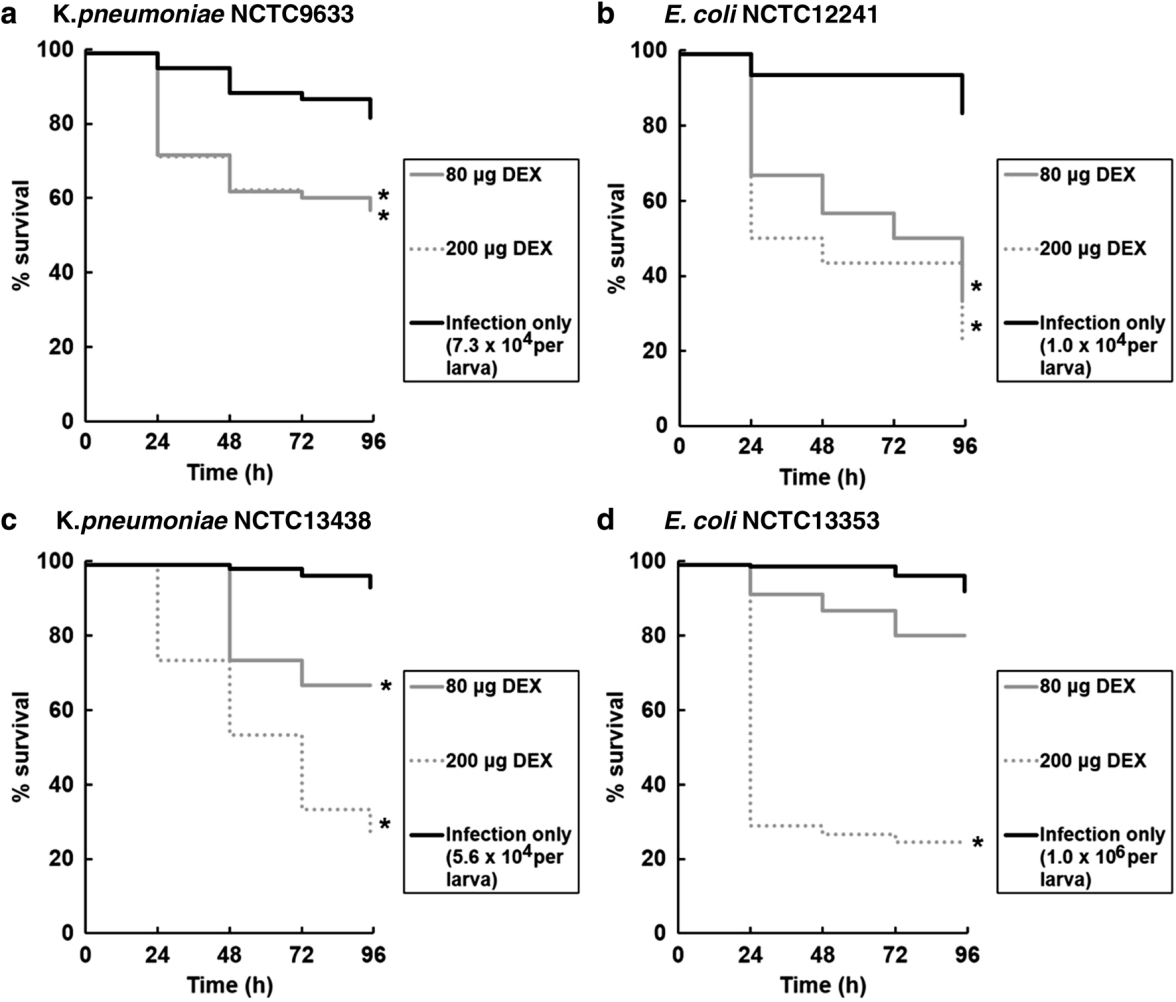
Effect of an increasing dose of dexamethasone 21-phosphate (DEX; 80 or 200 μg per larva) on the survival of *G. mellonella* larvae infected with *K. pneumoniae* NCTC9633 (**a**) or NCTC13438 (**c**) and *E. coli* NCTC12241 (**b**) or NCTC13353 (**d**) during incubation at 37 °C for 96 h. Numbers in the legend indicate the bacterial inoculum size in cells per larva. The control treatment groups labelled ‘infection only’ were injected with sterile water in place of dexamethasone 21-phosphate. Dexamethasone 21-phosphate or sterile water was administered to the larvae 10 min prior to infection with live bacteria. *Asterisk* indicates a group with significantly reduced survival compared to the control treated with water (*p* < 0.05, log-rank test with Holm correction for multiple comparisons); *n* = 30 (pooled from duplicate experiments)

Trial experiments determined the optimal exposure time and doses of dexamethasone 21-phosphate (data not shown). The effect of a 10-min exposure to 80 and 200 μg per larva doses of dexamethasone 21-phosphate prior to bacterial infection with *E. coli* or *K. pneumoniae* on survival of *G. mellonella* larvae is shown in Fig. 2. For each bacterial strain tested, deliberately small inoculum numbers were selected for infection to easily identify any increases in lethality that occurred due to exposure to dexamethasone. For all strains, prior treatment with dexamethasone, compared with sterile water, resulted in significantly increased lethality of infection of an identical inoculum. The increase in mortality also occurred in a dose-dependent manner for all strains, apart from infection with *K. pneumoniae* NCTC9633, where the higher dose of 200 μg per larva dexamethasone induced no additional larval mortality over that induced by the 80 μg per larva dose.

**Fig. 3:**
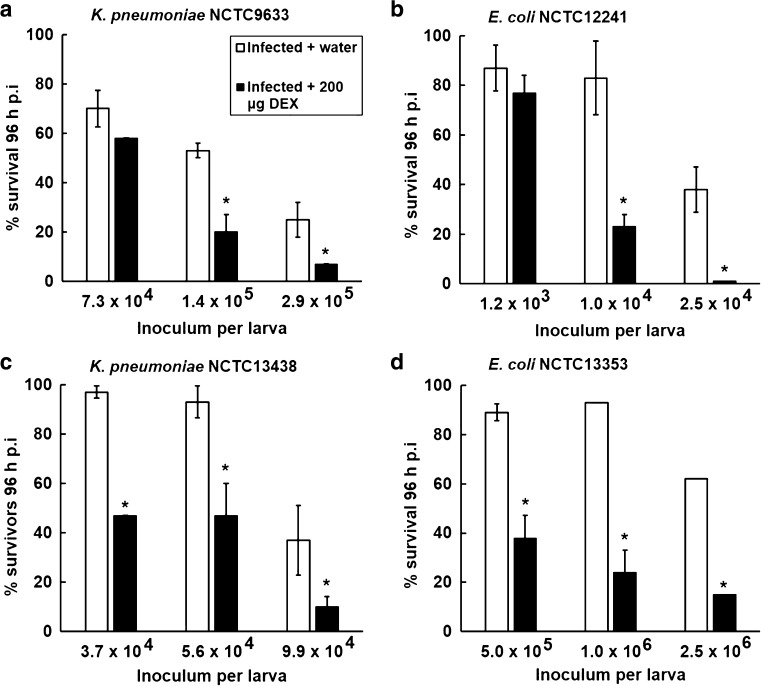
Effect of pre-treatment with dexamethasone 21-phosphate (DEX; 200 μg per larva) and subsequent infection with increasing inoculum sizes of *K. pneumoniae* NCTC9633 (**a**) or NCTC13438 (**c**) and *E. coli* NCTC12241 (**b**) or NCTC13353 (**d**) on the survival of *G. mellonella* larvae after 96 h at 37 °C. Groups treated with dexamethasone 21-phosphate were compared to controls treated with sterile water. Dexamethasone 21-phosphate or water was administered to the larvae 10 min prior to infection with live bacteria. *Asterisk* indicates a group with significantly reduced survival compared to the control treated with water (*p* < 0.05, log-rank test with Holm correction for multiple comparisons); *n* = 30 (pooled from duplicate experiments). ±Standard deviation error bars are shown for each treatment group

Following this, experiments determined the effect of increasing the inoculum size of infecting bacteria after prior treatment (10 min) with a fixed dose of dexamethasone 21-phosphate (Fig. 3). As expected, larval groups exposed to water, or dexamethasone (200 μg per larva), for 10 min prior to infection showed significantly increased mortality in a dose-dependent manner as the inoculum size of all strains increased. Notably, this inoculum-size-dependent lethality was significantly enhanced in larvae that had been pre-treated with dexamethasone (200 μg per larva) for 10 min prior to infection compared to the control groups treated with sterile water.

Importantly, at the concentrations tested, dexamethasone 21-phosphate treatment alone had no detrimental effects on *G. mellonella* larvae, thus ruling out the possibility that the enhanced lethality of infection induced by treatment with the compound could be explained by a non-specific toxic effect (data not shown; *p* > 0.05, log-rank test).

**Fig. 4:**
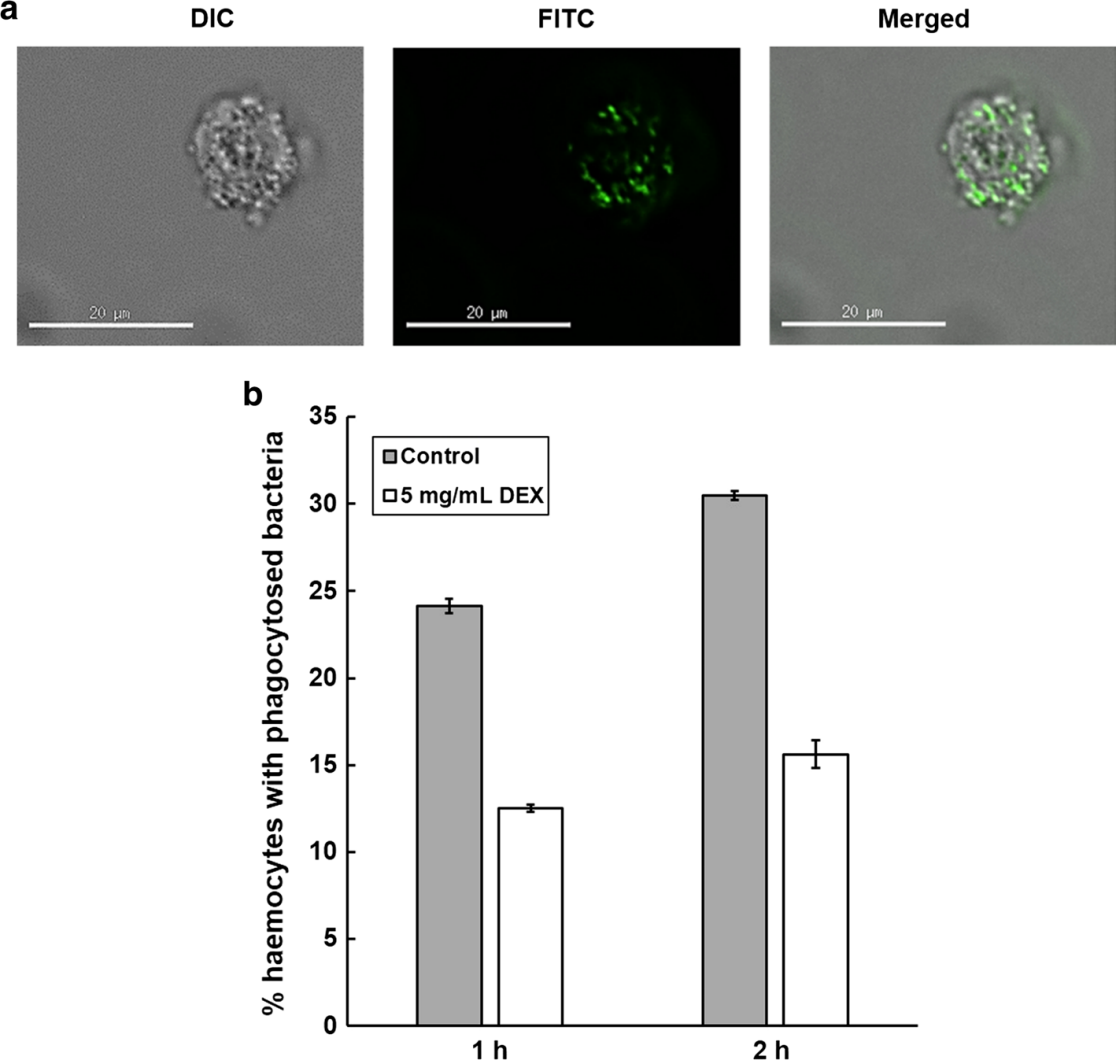
**a** Representative image of a single *G. mellonella* haemocyte after 1 h incubation at 37 °C with inactivated FITC-labelled *E. coli* K12. Images are shown with differential interference contrast (DIC), filters for FITC (excitation 490 nm/emission 528 nm) and a merged DIC/FITC image. The merged image represents a central 0.2 μM optical slice through the haemocyte allowing precise counting of internalised bacteria. Haemocytes and fluorescent *E. coli* were viewed at 40× magnification. **b** Effect of a 1- or 2 h incubation at 37 °C with dexamethasone 21-phosphate (DEX; 5 mg/mL) on the phagocytosis of inactivated fluorescein (FITC)-labelled *E. coli* K12 by purified haemocytes from *G. mellonella* larvae. Haemocytes and fluorescent *E. coli* were viewed using differential interference contrast or fluorescence microscopy at 40× magnification, and bacteria inside the haemocytes were counted. For each time point tested, phagocytosed *E. coli* from at least 30 haemocytes, from at least two replicate experiments, were counted per slide. ±SE bars are drawn for each treatment group

To confirm that the enhanced lethality of infection induced by exposure to dexamethasone 21-phosphate was due to a specific immunosuppressive effect, and whether the compound inhibits phagocytosis in *G. mellonella* similar to pure dexamethasone [20], phagocytosis assays were carried out. The effect of a 1- or 2 h incubation with 5 mg/mL dexamethasone 21-phosphate on phagocytosis in vitro of FITC-labelled *E. coli* by *G. mellonella* haemocytes is shown in Fig. 4. This concentration was selected after trial experiments with a range of concentrations (data not shown).

Figure 4a shows a representative image of a single *G. mellonella* haemocyte with phagocytosed FITC-labelled *E. coli* K12 taken after 1 h incubation at 37 °C. Bacteria are visible inside the haemocyte and were counted after studying images of optical slices through the haemocyte. In control experiments (treated with water), the proportion of haemocytes that contained phagocytosed *E. coli* was approximately 25 % after 1 h and 30 % after 2 h (Fig. 4b). Exposure to 5 mg/mL dexamethasone 21-phosphate reduced this to approximately 12 and 15 % after 1 and 2 h, respectively (Fig. 4b). The observed inhibition of phagocytosis was not due to a toxic effect of dexamethasone 21-phosphate because measurement of haemocyte viability revealed no significant detrimental effect of exposure to the compound under the conditions tested (data not shown).

**Fig. 5:**
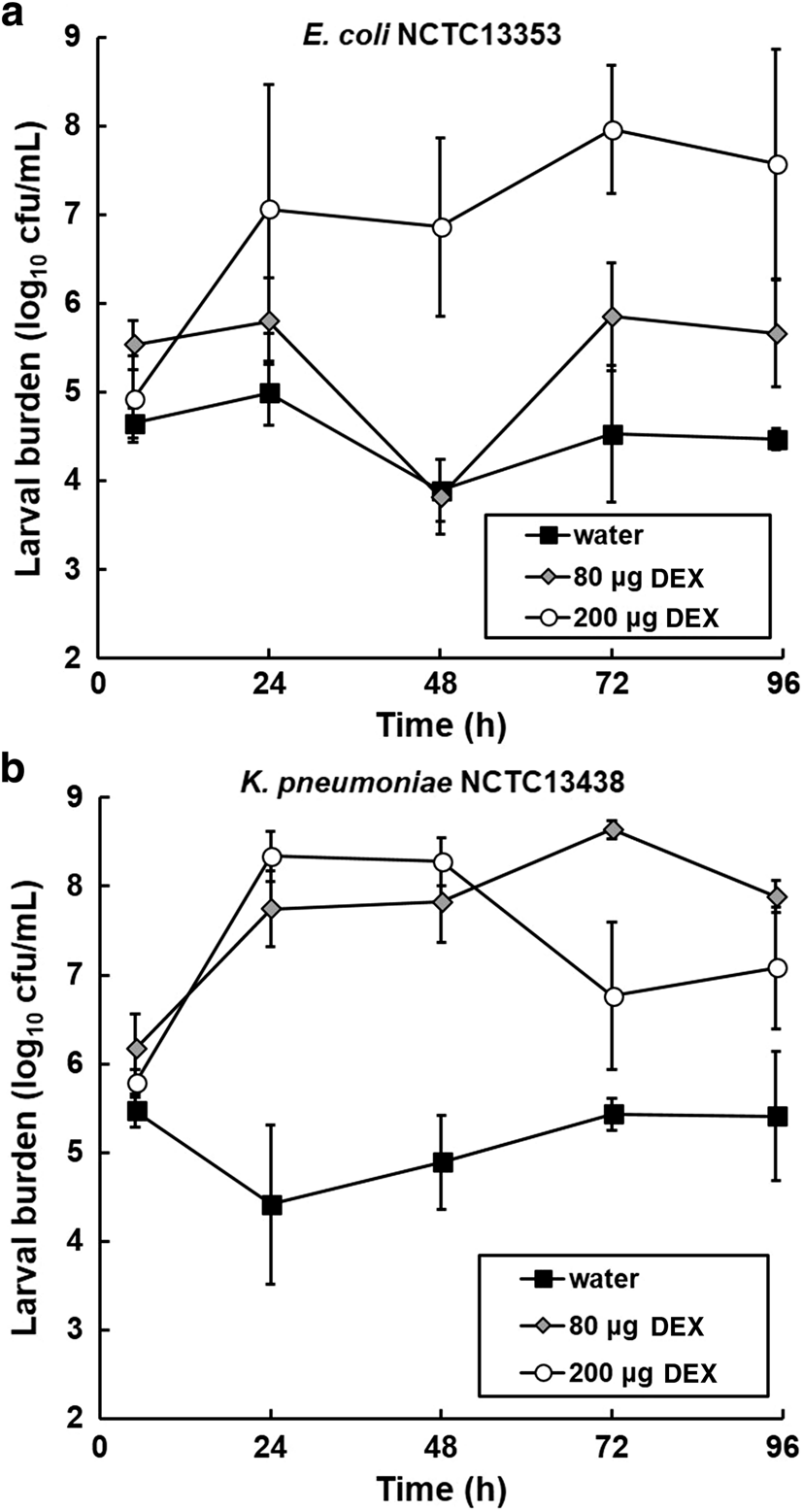
Effect of pre-treatment with dexamethasone 21-phosphate (DEX; 80 or 200 μg per larva) and subsequent infection with 1.0 × 10^6^ cells per larva of *E. coli* NCTC13353 (**a**) or 5.6 × 10^4^ cells per larva of *K. pneumoniae* NCTC13438 (**b**) on larval burden of bacteria during incubation at 37 °C for 96 h. Larvae treated with dexamethasone 21-phosphate were compared to controls treated with sterile water. Dexamethasone 21-phosphate or water was administered to the larvae 10 min prior to infection with live bacteria. Larval burden was determined from 5 individual larvae every 24 h. The data shown is from duplicate experiments and the error bars indicate the ± standard error

The effect of immunosuppressive treatment with dexamethasone 21-phosphate on the proliferation of bacteria in *G. mellonella* larvae over 96 h was measured via a bacterial burden assay (Fig. 5). Inoculum sizes of *E. coli* NCTC13353 (1.0 × 10^6^ cells per larva) and *K. pneumoniae* NCTC13438 (5.6 × 10^4^ cells per larva) were chosen that induced large differences in levels of mortality in populations that were pre-treated with water compared to 200 μg per larva of dexamethasone 21-phosphate (Fig. 3). In control populations of larvae, infected with either the *E. coli* or the *K. pneumoniae* strain after pre-treatment with water, the bacterial population size did not increase over the 96 h duration of the experiment (Fig. 5). This implies that the innate immune system of the larvae prevents the bacteria from proliferating and reflects the low levels of mortality observed under these conditions (Fig. 3). In contrast, pre-treatment with 80 or 200 μg per larva of dexamethasone prior to infection resulted in significant increases in bacterial numbers, of either the *E. coli* or the *K. pneumoniae* strain, inside the larvae after just 24 h (Fig. 5). Post-24 h, there is some fluctuation in total bacterial numbers detected that may be due to the random sampling of the five larvae from the total population for each time point. Nonetheless, this proliferation of bacteria correlated with the enhanced mortality of infected larvae induced upon exposure to an identical dose of dexamethasone observed previously (Fig. 3).

To conclude, treatment of *G. mellonella* larvae with dexamethasone 21-phosphate enhanced the lethality induced by infection with *E. coli* or *K. pneumoniae* in a dose- and inoculum size-dependent manner. The enhanced lethality observed correlated with proliferation of bacteria in the larvae that could be attributed to dexamethasone inhibiting haemocyte phagocytosis and thus acting as an immunosuppressant.

### The efficacy of antibiotic therapy versus *K. pneumoniae* or *E. coli* infection is reduced in larvae treated with dexamethasone 21-phosphate

**Fig. 6:**
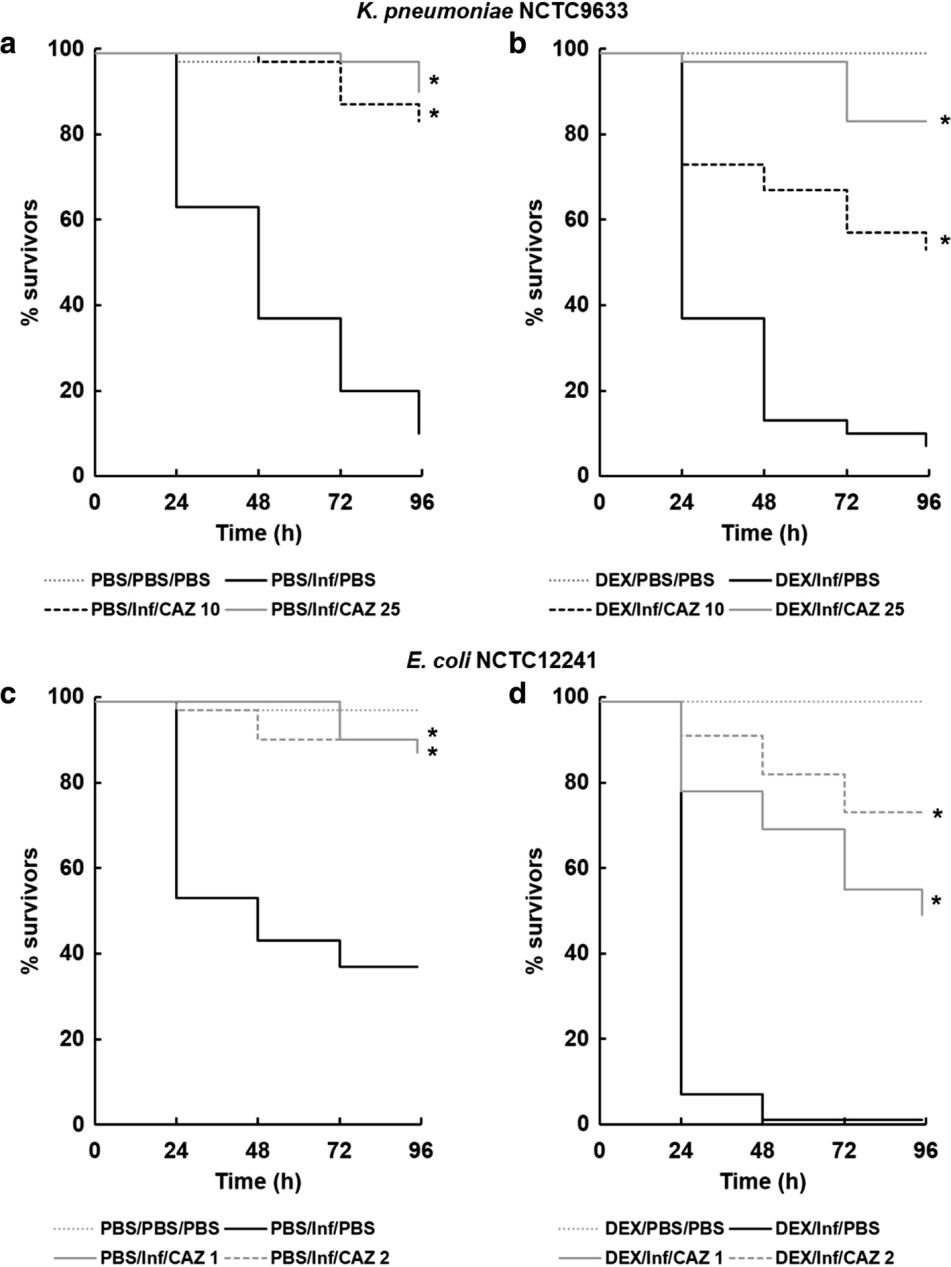
Effect of ceftazidime treatment on survival of *G. mellonella* larvae pre-exposed to dexamethasone 21-phosphate (DEX; 200 μg per larva) prior to infection with 2.9 × 10^5^ cells per larva of *K. pneumoniae* NCTC9633 (**a**, **b**), or 2.5 × 10^4^ cells per larva of *E. coli* NCTC12241 (**c**, **d**). *E. coli* NCTC12241 and *K. pneumoniae* NCTC9633 do not possess β-lactamases and were sensitive to ceftazidime in vitro with MIC values of 0.25 and 0.5 mg/L, respectively. Survival was measured during incubation at 37 °C for 96 h, and dexamethasone 21-phosphate-treated groups (**b**, **d**) were compared to controls treated with water (**a**, **c**). Dexamethasone 21-phosphate or water was administered to the larvae 10 min prior to infection with live bacteria. A single dose of ceftazidime (CAZ) was administered 2 h post-infection: 10 and 25 mg/kg for *K. pneumoniae* NCTC9633 (**a**, **b**), and 1 and 2 mg/kg for *E. coli* NCTC12241 (**c**, **d**). *Asterisk* indicates a group with significantly enhanced survival compared to the infected control treated with PBS (*p* < 0.05, log-rank test). *n* = 30 (pooled from duplicate experiments)

The MICs of ceftazidime for the four strains used in this work were: *E. coli* NCTC12241 and NCTC13353, 0.25 and 64 mg/L, respectively; and *K. pneumoniae* NCTC9633 and NCTC13438, 0.5 and ≥512 mg/L, respectively. The effect of treatment with ceftazidime, with or without prior exposure to dexamethasone-21-phosphate, on larvae infected with the two sensitive strains, *E. coli* NCTC12241 and *K. pneumoniae* NCTC9633, is shown in Fig. 6. 2 h post-infection, the larvae were treated with either PBS or increasing doses of ceftazidime (10 or 25 mg/kg for *K. pneumoniae* NCTC9633; and 1 or 2 mg/kg for *E. coli* NCTC12241). Supporting the previous observations, in the control groups administered PBS rather than the antibiotic, exposure to dexamethasone 21-phosphate (200 μg per larva) prior to infection resulted in enhanced lethality of both strains compared to treatment with water. However, in control groups treated with water in the absence of dexamethasone 21-phosphate, administration of ceftazidime significantly improved survival of larvae, infected with either strain, in a dose-dependent manner. In comparison, after prior exposure to dexamethasone 21-phosphate, the efficacy of identical doses of ceftazidime was significantly reduced.

In summary, prior exposure to the immunosuppressant dexamethasone 21-phosphate not only enhances *G. mellonella* lethality induced by infection with *E. coli* or *K. pneumoniae* but also reduces the efficacy of a normally effective antibiotic treatment.

## Discussion

The sensitivity of *G. mellonella* larvae to infection by the *E. coli* or *K. pneumoniae* strains in this work compares favourably with that identified in previous studies [8–11, 13–15]. For example, between 1.0 × 10^5^ and 5.0 × 10^6^ CFU per larva are required for *E. coli* to induce significant mortality [13–15] and between 1.0 × 10^5^ and 1.0 × 10^6^ CFU per larva for *K. pneumoniae* [8–11]. In contrast, different strains of *Pseudomonas aeruginosa* induced significant lethality of *G. mellonella* after infection with as few as 25 cells per larva [16, 17]. This implies that either the innate immune system of *G. mellonella* is better adapted to combat *K. pneumoniae* and *E. coli*, or these pathogens are less virulent than *P. aeruginosa* in the larvae, or a combination of both factors could contribute to the observed differences.

In response to bacterial infection, the immune system of *G. mellonella* forms melanotic nodules whose formation is regulated by signalling molecules termed eicosanoids. Nodules form when insect haemocytes degranulate upon contact with bacteria followed by activation of the prophenoloxidase cascade which results in the formation of melanin around the bacteria. Subsequently, a capsule forms around the sequestered bacteria in the developing nodule due to attachment and spreading of further haemocytes followed by the commencement of phagocytic activity to remove and destroy the bacteria [21]. Mandato et al. [20] showed that the eicosanoid biosynthesis inhibitor dexamethasone inhibited prophenoloxidase activation in vivo and haemocyte spreading and phagocytosis in vitro. Supporting this, a recent study showed that administration of dexamethasone repressed *G. mellonella* innate immunity in a similar fashion to injected entomopathogenic nematode cuticles [22].

This work has shown that the anti-inflammatory drug dexamethasone 21-phosphate also acts as an immunosuppressant in *G. mellonella* larvae. Larvae exposed to the drug become more sensitive to infection by *E. coli* and *K. pneumoniae* and die more rapidly than untreated equivalents. This enhanced lethality occurs due to the uncontrolled proliferation of bacteria within the infected larvae that can be at least partially attributed to the inhibitory effect treatment with the drug has on haemocyte phagocytosis. Supporting these findings, studies using the alternative immunosuppressant drugs, cyclosporin A [23] and hydrocortisone [24], also enhanced the lethality of bacterial infections, by *Pseudomonas aeruginosa* and *Enterobacter cloacae*, respectively, in *G. mellonella* larvae.

Dexamethasone 21-phosphate has the advantage of being water soluble and easier to administer then dexamethasone which is only soluble at use concentrations in organic solvents, such as ethanol or dimethyl sulphoxide, which, in our hands, had toxic side effects on *G. mellonella* larvae (data not shown).

Doses of dexamethasone phosphate used in humans are variable depending on the underlying condition and severity of disease. For sepsis, doses between 0.3 and 30 mg/kg have been administered in a range of published trials (reviewed in [25]). The highest effective dose administered in this work (200 μg per larva) equates to a dose of 800 mg/kg, indicating that the immunosuppressive effect of dexamethasone phosphate is far more potent in human patients than in *G. mellonella* larvae.

Successful application of an immunosuppressant with the *G. mellonella* infection model is significant because immunosuppression may facilitate studies on infection and treatment of alternative human pathogens that have low virulence or do not normally induce acute infections in this invertebrate [26]. Furthermore, the use of immunosuppression will better mimic the physiological state of many human patients that suffer from hospital-acquired infections caused by organisms such as *E. coli* and *K. pneumoniae* and thus improve the clinical relevance of this invertebrate model. Finally, this work has shown that immunosuppression can be used to improve the specificity of infection in *G. mellonella*. For example, as discussed earlier, infections with *E. coli* and *K. pneumoniae* require high inoculum numbers to induce lethality of the larvae. As a consequence of this, infections with identical inocula that were heat-killed induced high levels of larval mortality that could not be attributed solely to infection by live bacteria but were also due to the presence of toxic components of the dead bacteria. Treatment with dexamethasone 21-phosphate allowed infections with smaller inoculum sizes of both *E. coli* and *K. pneumoniae* to induce substantial larval lethality where equivalent heat-killed inocula did not induce significant pathogenicity. This implies that the specificity of infection of *G. mellonella* can be significantly improved by using dexamethasone phosphate to alleviate the need to use high cell number inocula of bacterial species such as *E. coli* and *K. pneumoniae*. A possible explanation for the observed toxicity of the dead cells may be due to the induction of a pro-inflammatory effect analogous to that seen with Gram-negative sepsis in humans, but this would require additional investigation that is outside the scope of this study.

Distinct from administering a specific drug to induce immunosuppression, but corroborating this finding, enhanced susceptibility of *G. mellonella* larvae to microbial infection has been reported after exposure to different stress factors that impair aspects of innate immunity. For example, infection by *Candida albicans* or *Staphylococcus aureus* induced greater larval mortality after prolonged incubation over a 10-week period at 15 °C that was attributed to a combination of a decline in certain metabolic pathways and changes in the relative abundance of haemocytes [27]. Similarly, nutrient deprivation of larvae also has immunosuppressive effects and results in lower haemocyte density and a reduction in the expression of antimicrobial peptides leading to increased susceptibility to *C. albicans* infection [28].

In the clinical setting, the co-administration of anti-inflammatory drugs with antibiotics remains controversial. Adjunctive treatment with corticosteroids such as dexamethasone is used to decrease inflammation and mortality in bacterial meningitis or sepsis [25, 29]. However, evidence has been presented that this could contribute to failure of antibiotic therapy [30, 31]. In this work, administration of ceftazidime to *G. mellonella* larvae infected with susceptible strains of *E. coli* and *K. pneumoniae* resulted in enhanced survival. However, the efficacy of the antibiotic was reduced in immunosuppressed larvae that had been treated with dexamethasone 21-phosphate prior to infection. This could be explained by the immunosuppressant drug interfering with the inhibitory action of the antibiotic, or because the immunosuppressant inhibits aspects of innate immunity, and as a consequence allows the infecting bacteria to proliferate (as shown in this study), an identical dose of antibiotic would not be as efficacious as it would be in larvae not immunosuppressed. Alternatively, dexamethasone can induce overexpression of *tolC*, repression of OmpF, stimulate antibiotic efflux and ultimately reduce the susceptibility of *E. coli* to antibiotics in vitro [32].

The reduction in antibiotic efficacy seen with immunosuppressive therapy merits further investigation with the *G. mellonella* model to help clarify the relationship between the immune system and antibiotic treatment to clearance of bacteria and the relative contributions of each to abrogation of infection. Notably, this could have some clinical relevance to the adjunctive use of anti-inflammatory drugs with antibiotics that is used to tackle some serious bacterial infections.

To conclude, the demonstration of an effective immunosuppressant regimen in the *G. mellonella* infection model shown here should facilitate additional studies on novel treatments to combat MDR Gram –ve bacteria, such as *E. coli* and *K. pneumoniae*, that cause serious infections in hospitals caring for already severely ill patients and further develops *G. mellonella* as a serious alternative model host.
